# Topical pharyngeal anesthesia provides no additional benefit to propofol sedation for esophagogastroduodenoscopy: a randomized controlled double-blinded clinical trial

**DOI:** 10.1038/s41598-018-25164-7

**Published:** 2018-04-27

**Authors:** Xiaotian Sun, Yang Xu, Xueting Zhang, Aitong Li, Hanqing Zhang, Teng Yang, Yan Liu

**Affiliations:** 1 Department of Gastroenterology, 307 Hospital, Beijing, 100071 China; 2 Department of Internal Medicine, Clinic of August First Film Studio, Beijing, 100161 China

## Abstract

Propofol sedation has been applied during esophagogastroduodenoscopy procedures, but whether topical pharyngeal anesthesia should be administered at the same time has rarely been reported. Our study examined the role of topical pharyngeal anesthesia in sedated endoscopies in a randomized controlled double-blinded clinical trial. A total of 626 patients who underwent sedated esophagogastroduodenoscopy were randomized into the experimental group (n = 313) or the control group (n = 313). The discomfort score, immediately and one day after the procedure, was not statistically significant [7.2 (5–9) vs. 7.5 (6–9), P = 0.210; 2.3 (0–3) vs. 2.6 (0–4), P = 0.095, respectively]. Two patients in the experimental group and three patients in the control group needed oral medication for pharyngeal discomfort (P = 0.354). The satisfaction score was 9.2 (8–10) in the experimental group and 8.9 (7–10) in the control group (P = 0.778). Lidocaine topical pharyngeal anesthesia in propofol-sedated esophagogastroduodenoscopy did not further reduce the pharyngeal discomfort or improve the satisfaction. This clinical trial was registered at ClinicalTrials.gov (ClinicalTrials.gov ID: NCT03070379).

## Introduction

Esophagogastroduodenoscopy is a very important tool for diagnosing and treating upper gastrointestinal diseases^1^. Recently, the number of patients who undergo sedated esophagogastroduodenoscopy has been increasing^2,3^. Propofol sedation has been widely introduced in sedated endoscopic examinations, which has the advantages of less adverse events, high satisfaction, less discomfort and simple administration^3–5^. For those sedated patients, whether lidocaine topical pharyngeal anesthesia should be administered is still in doubt^6^, although lidocaine topical pharyngeal anesthesia as a routine pretreatment for esophagogastroduodenoscopy may facilitate the intubation of the endoscopy and reduce the injury of the pharyngeal mucosa^7^. The European Society of Gastrointestinal Endoscopy, the European Society of Gastroenterology and Endoscopy Nurses and Associates, and the European Society of Anesthesiology Guidelines for the non-anesthesiologist administration of propofol for gastrointestinal endoscopy have not made any specific recommendations on this topic^8,9^, mainly because the role of pharyngeal anesthesia during propofol sedation for upper digestive endoscopy has not been completely clarified^10,11^.

Considering the fact that lidocaine anesthesia may cause airway narrowing and anaphylaxis, it is important to examine the role of lidocaine topical pharyngeal anesthesia in esophagogastroduodenoscopy under propofol sedation, which has rarely been reported in large-scale clinical trials so far; the findings have not quite been consistent among different studies^10,12^. Thus, in this study, we test whether lidocaine topical pharyngeal anesthesia should be performed in propofol-sedated esophagogastroduodenoscopy in a randomized controlled trial, aiming at investigating whether lidocaine topical pharyngeal anesthesia could benefit patients who underwent esophagogastroduodenoscopy under propofol sedation.

## Methods

### Patients

Consecutive outpatients who had an indication for esophagogastroduodenoscopy and planned to undergo sedated endoscopic examination (n = 636) from February 2017 to May 2017 in the endoscopy center of 307 Hospital were enrolled. Those who were unwilling to participate in this study (n = 10) were excluded. The demographic and clinical data were retrieved and collected from the computerized database. The main indications for esophagogastroduodenoscopy included heartburn, abdominal distension, dyspepsia and epigastric pain. The endoscopic diagnosis included esophagitis, chronic gastritis, peptic ulcer, esophageal cancer and gastric cancer. Only one primary indication and one endoscopic diagnosis were recorded based on the severity.

All patients signed a written informed consent. This study was approved by the Ethics Committee of 307 Hospital of Academy of Military Medical Science, and the study design and protocol were in accordance with the Declaration of Helsinki.

### Study design

**Figure 1:**
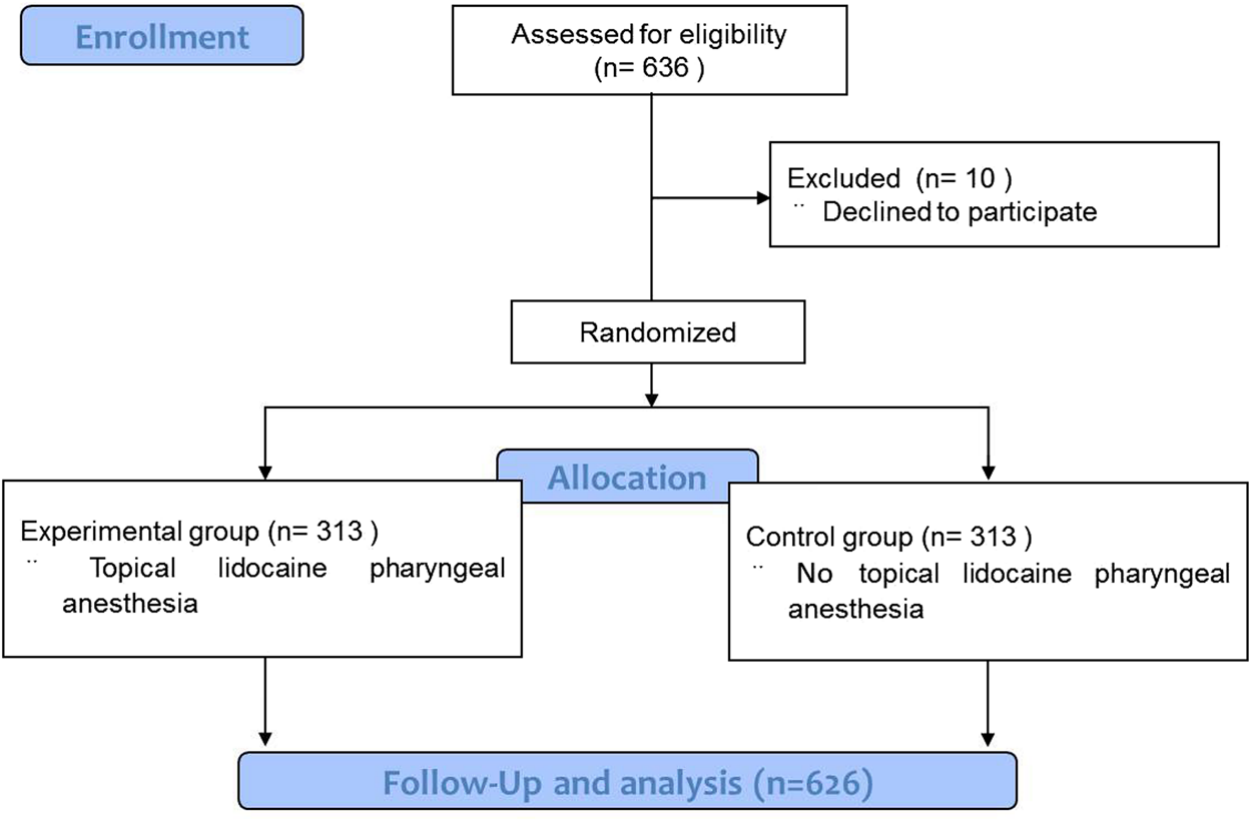
Flowchart of the study.

This study was designed as a randomized controlled double-blinded clinical trial (Fig. 1). The sample size was calculated based on a probability of 0.8 and α error of 0.05. The required sample size was set to 600 patients, and 626 patients were enrolled. All patients were randomly divided into two groups in the ratio of 1:1 using the random number method. In the experimental group (n = 313), topical pharyngeal anesthesia by 2% lidocaine spray was administered by the endoscopic nurses 3 times 4–5 minutes before propofol sedation in patients who underwent esophagogastroduodenoscopy. These 3 sprays of lidocaine totaled approximately 3 ml. In the control group (n = 313), lidocaine was not administered, and standard saline solution was sprayed. Patients and endoscopists were blinded to the grouping information. The data were collected and analyzed by two independent investigators. This clinical trial was registered at ClinicalTrials.gov (ClinicalTrials.gov ID: NCT03070379) on March 2, 2017.

### Esophagogastroduodenoscopy

All procedures were completed by one endoscopist with over 3-years of endoscopy experience. Propofol sedation was administered by one anesthetist. EG-L590WR endoscopes equipped with the LASEREO endoscopic system (FUJIFILM Co., Tokyo, Japan) were used. After the procedure, all patients were monitored for 1 hour.

### Outcomes

The primary outcome measures were immediate throat discomfort and satisfaction. The secondary outcome measures included throat discomfort 1 day after the procedure, oral medication and adverse events. It is anticipated that the use of topical pharyngeal anesthesia may greatly reduce the throat discomfort in patients who undergo sedated esophagogastroduodenoscopy. The discomfort score was evaluated using the Visual Analogue Scale (VAS) method with 0 as no discomfort and 10 as maximal discomfort. The satisfaction score was judged using VAS with 0 as minimal satisfaction and 10 as maximal satisfaction. The VAS was ordinal, meaning the patients could only choose whole numbers on the scale.

### Follow-up

All patients were followed up by telephone after 1 day of the endoscopic examination. The discomfort score was calculated based on VAS. No patients were lost to follow-up, and the follow-up rate was 100%.

### Statistical analysis

All statistical analysis were conducted using SPSS 17.0 software (SPSS Inc, Chicago, USA). Categorical and continuous data were presented as percentage (%) and mean (range), and the differences were tested by the chi-square test and Wilcoxon-Mann-Whitney test when applicable. A two-tailed P value less than 0.05 was considered to be statistically significant.

## Results

### Demographic and clinical characteristics

No statistically significant differences were found on the age, gender, previous esophagogastroduodenoscopy, history of pharyngeal diseases and smoking >5 years between the experimental and control group (all P values > 0.05, Table 1).

**Table 1:** Demographic and clinical characteristics.

	Experimental group (n = 313)	Control group (n = 313)	P value
Age, years	21–56	20–60	0.122
Gender, n (%)			0.143
Female	119 (38.0)	138 (44.1)	
Male	194 (62.0)	175 (55.9)	
Previous esophagogastroduodenoscopy, n (%)	105 (33.5)	99 (31.6)	0.501
History of pharyngeal diseases, n (%)	201 (64.2)	203 (64.8)	0.554
Smoking >5 years, n (%)	173 (55.3)	169 (54.0)	0.290
Main indications, n (%)			0.718
Heartburn	118 (37.7)	96 (30.7)	
Abdominal distension	57 (18.2)	66 (21.1)	
Dyspepsia	92 (29.4)	93 (29.7)	
Epigastric pain	46 (14.7)	58 (18.5)	
Endoscopic diagnosis, n (%)			0.249
Esophagitis	47 (15.0)	42 (13.4)	
Chronic gastritis	221 (70.6)	231 (73.8)	
Peptic ulcer	33 (10.5)	27 (8.6)	
Esophageal cancer	4 (1.3)	3 (1.0)	
Gastric cancer	8 (2.6)	10 (3.2)	

The main indications for endoscopic examination in the experimental group included heartburn in 118 patients (37.7%), abdominal distension in 57 patients (18.2%), dyspepsia in 92 patients (29.4%) and epigastric pain in 46 patients (14.7%); in the control group, the main indications for endoscopic examination included heartburn in 96 patients (30.7%), abdominal distension in 66 patients (21.1%), dyspepsia in 93 patients (29.7%) and epigastric pain in 58 patients (18.5%); there were no significant differences between the two groups (P = 0.718). Endoscopic diagnosis included esophagitis in 47 patients (15.0%), chronic gastritis in 221 patients (70.6%), peptic ulcer in 33 patients (10.5%), esophageal cancer in 4 patients (1.3%) and gastric cancer in 8 patients (2.6%) in the experimental group; in the control group, esophagitis was diagnosed in 42 patients (13.4%), chronic gastritis in 231 patients (73.8%), peptic ulcer in 27 patients (8.6%), esophageal cancer in 3 patients (1.0%) and gastric cancer in 10 patients (3.2%); overall, there were no significant differences (P = 0.249). The dosage of propofol used for the procedures was comparable between the experimental and control groups [140 (100–170) mg vs. 145 (100–175) mg; P = 0.311, respectively].

### Discomfort and satisfaction evaluation

The examination duration was 13–18 minutes in the experimental group and 12–18 minutes in control group, which was comparable between the two groups (P = 0.344, Table 2). The discomfort score immediately and one day after the procedure was 7.2 (5–9) and 2.3 (0–3) in the experimental group, respectively, and 7.5 (6–9) and 2.6 (0–4) in the control group (P = 0.210, P = 0.095). Two patients (0.3%) in the experimental group and three patients (0.5%) in the control group needed oral medication for pharyngeal discomfort (P = 0.354). The satisfaction score with the procedure was 9.2 (8–10) in the experimental group and 8.9 (7–10) in the control group (P = 0.778).

**Table 2:** Discomfort score evaluation.

	Experimental group (n = 313)	Control group (n = 313)	P value
Examination duration, min	13–18	12–18	0.344
Discomfort score, mean (range)			
Immediately after procedure	7.2 (5–9)	7.5 (6–9)	0.210
One day after procedure	2.3 (0–3)	2.6 (0–4)	0.095
Oral medication needed for pharyngeal discomfort, n (%)	2 (0.3)	3 (0.5)	0.354
Satisfaction score with the procedure, mean (range)	9.2 (8–10)	8.9 (7–10)	0.778

## Discussion

The role of esophagogastroduodenoscopy in diagnosing and treating upper digestive mucosal lesions has been well acknowledged, especially with the advancement of endoscopic imaging techniques^13,14^. However, the discomfort of the intubation during the sedated procedure may impair the patients’ compliance, especially if there is an injury to the pharyngeal mucosa^15,16^. Thus, topical lidocaine pharyngeal anesthesia is a routine procedure, which gives the advantage of lubrication and less discomfort^17^. For patients who undergo esophagogastroduodenoscopy under propofol sedation, whether topical pharyngeal anesthesia is needed remains a controversial topic^18^. Additionally, the current guidelines on sedated endoscopy did not make any suggestions on this topic, which should be further examined in clinical research^8^. Thus, we designed and conducted this randomized controlled double-blinded clinical trial to investigate and compare the experimental group and control group regarding discomfort and satisfaction evaluation during and after the esophagogastroduodenoscopic examinations. These results indicated that the additional administration of lidocaine topical pharyngeal anesthesia in sedated esophagogastroduodenoscopy did not further reduce the pharyngeal discomfort and improve the satisfaction during the procedure.

In this study, consecutive outpatients who underwent sedated esophagogastroduodenoscopy were enrolled and randomly divided into the experimental and control groups. The gender, age, and main indications for endoscopic examination and endoscopic diagnosis between the two groups were comparable (all P values > 0.05). Heartburn was the most common primary indication for esophagogastroduodenoscopy in both groups, and there were 221 patients (70.6%) in the experimental group and 231 patients (73.8%) in the control group who were diagnosed with chronic gastritis. We also observed that the topical pharyngeal anesthesia did not significantly reduce the discomfort score both immediately and one day after the endoscopic examinations [7.2 (5–9) vs. 7.5 (6–9), P = 0.210; 2.3 (0–3) vs. 2.6 (0–4), P = 0.095, respectively], making our results consistent with a previous study^19^. However, it was reported that lidocaine is the ideal pharyngeal anesthetic to ensure the adaptation of the patient to the procedure and to decrease anxiety and discomfort during the procedure^7^. A lack of standardized outcome measurements and standardized sedation strategies may be the reason for the heterogeneity of the conclusions among different studies^10^. Furthermore, few patients reported severe pharyngeal discomfort or needed oral medication, and thus, we did not conduct subgroup analysis based on the age, gender, smoking and other demographic and clinical characteristics. Nonetheless, through an unsystematic data review, we found that 5/5 patients who took oral medication also had pharyngeal disease. It was supposed that for such patients with pharyngeal diseases, topical pharyngeal anesthesia might be recommended to reduce injury to the local mucosa caused by the intubation of the esophagogastroduodenoscopy. However, topical pharyngeal anesthesia did not significantly decrease the incidence of severe pharyngeal discomfort after esophagogastroduodenoscopy (2/201 vs. 3/203) based on our data. Furthermore, there may also be other unaccounted variables to be investigated.

There were still some limitations in this study. First, all patients were from one center. A multicenter clinical trial could be planned for further validation. Second, multivariate analysis of the discomfort or noncompliance with esophagogastroduodenoscopy was not performed. This was not done because the percentage of patients with severe discomfort was very low. Third, lidocaine was used for topical anesthesia in this study because lidocaine is commonly used in clinical practice, while previous studies also compared other medications for topical anesthesia^20–22^, which may be further assessed in future.

Our study provided evidence that helps prove that lidocaine as a topical pharyngeal anesthetic was not a necessity for propofol-sedated esophagogastroduodenoscopy and does not need to be routinely performed before propofol sedation. For patients with pharyngeal diseases such as chronic pharyngitis, topical lidocaine pharyngeal anesthesia conferred no obvious benefits.
